# Use of eschar swabbing for the molecular diagnosis and genotyping of *Orientia tsutsugamushi* causing scrub typhus in Quang Nam province, Vietnam

**DOI:** 10.1371/journal.pntd.0005397

**Published:** 2017-02-27

**Authors:** Nhiem Le Viet, Maureen Laroche, Hoa L. Thi Pham, Nho L. Viet, Oleg Mediannikov, Didier Raoult, Philippe Parola

**Affiliations:** 1 URMITE, Aix Marseille Université, UM63, CNRS 7278, IRD 198, INSERM 1095, IHU - Méditerranée Infection, Marseille, France; 2 Department of Tropical Diseases, Quang Nam Central General Hospital, Quang Nam, Vietnam; 3 Department of Infectious Diseases, University of Medicine and Pharmacy of Ho Chi Minh City, Ho Chi Minh City, Vietnam; 4 Department of Internal Medicine, Quang Nam Central General Hospital, Quang Nam, Vietnam; University of Tennessee, UNITED STATES

## Abstract

**Background:**

Scrub typhus is a rickettsiosis which is caused by *Orientia tsutsugamushi* and occurs throughout the Asia-Pacific region. Molecular diagnosis of rickettsioses using eschar swabs has recently emerged, and may be very useful for the diagnosis of these diseases in tropical settings.

**Methodology/Principal findings:**

Quantitative polymerase chain reaction (qPCR) was used to detect *O*. *tsutsugamushi* DNA in whole blood and eschar swab specimens of 67 patients who were clinically suspected of scrub typhus in Quang Nam province, Vietnam. Among the 20 patients for whom both eschar and whole blood were obtained, 17 (85%) of the eschar specimens and 5 (25%) of the whole blood specimens tested positive for *O*. *tsutsugamushi*. Genetic analysis of the 56-kDa TSA gene sequences demonstrated that the 14 sequences obtained in this study, including 12 eschar swabs and 2 whole blood specimens, were related to 4 groups: Karp, Kawasaki, Gilliam (JG-v and TG-v) and TA716. The majority (9/14; 64.4%) of contemporary *O*. *tsutsugamushi* genotypes in Quang Nam province were related to the Karp group.

**Conclusions:**

These results suggest that polyclonal antigen pools used for serological testing in the future should contain at least Karp, Kawasaki, Gilliam and TA716 antigens for Vietnamese patients, as well as patients who have traveled to Vietnam. qPCR after eschar swabbing should be considered for molecular diagnosis of scrub typhus in endemic patients as well as in travelers, since it is easy to perform and appears very useful for the rapid detection of *Orientia tsutsugamushi* in the early phase of infection.

## Author summary

*Orientia tsutsugamushi* is the causative agent of scrub typhus, one of the most common of the rickettsioses in Pacific Asia. Although the disease is an important public health issue in Vietnam, there is a lack of diagnostic tools in almost all health facilities and very little clinical research has been done. In particular, the genotypes of the bacterium were not well known, with only one previous study performed in Vietnamese patients. We conducted this study in Quang Nam province, an endemic area in central Vietnam, for the first time using an eschar swab to detect the DNA of *O*. *tsutsugamushi* using molecular techniques. We also examined the genetic diversity of the bacteria based on sequencing, using the 56 k-Da TSA gene.

## Introduction

Scrub typhus is a rickettsiosis which is caused by *Orientia tsutsugamushi* (formerly named *Rickettsia tsutsugamushi*), transmitted by the bite of larval trombiculid mites [1, 2]. The disease is known to occur throughout the Asia-Pacific region but its range may be larger, with possible case reports in Africa [3] and South America [4, 5]. In Southeast Asia, it is thought that up to 1 million cases occur per year [6], and a significant proportion of hospital admissions for acute undifferentiated fever (AUF) are attributable to scrub typhus [4, 6–8]. Clinical manifestations can vary from mild to fatal disease in the absence of appropriate antibiotic treatment [9]. Treatment with either doxycycline or azithromycin is still highly effective for both children and adults, although evidence of *O*. *tsutsugamushi* resistance to doxycycline has been reported in northern Thailand [10]. Since there is no vaccine available, the main current prevention method is vector control and avoidance of exposure. Diagnosis of *O*. *tsutsugamushi* as well as the other *Rickettsia* species in human rickettsioses usually relies upon serology and molecular identification of the causative agent from blood or skin biopsy samples [11]. Serological evidence of infection generally appears in the second or third week of illness, and skin biopsy of an eschar is an invasive and potentially painful procedure; therefore, these are not always useful for clinical practice [12]. Following the description of the performance of eschar swabbing for the detection of *O*. *tsutsugamushi* and other Rickettsia species DNA on animals [12], other studies have validated this methods for humans [12–16].

*O*. *tsutsugamushi* contains many antigenic variants, including Gilliam, Kato, Karp, Kawasaki, Kuroki and other types [17, 18]. This antigenic variation depends largely on diversities of the immune-dominant 56-kDa type-specific antigen (TSA) located on the surface of the bacterial membrane [18, 19]. Sequencing of *O*. *tsutsugamushi* using this gene suggests that there is genetic diversity of the bacteria in Thailand [20], Taiwan [21], India [22], Cambodia [23], Laos [24] and China [25]. The variability of the 56-kDa TSA and its products could have an important role on the accuracy of diagnostic tests, vaccine development and epidemic disease control in endemic areas [20, 26].

In Vietnam, scrub typhus has been suggested to be one of the three major causes of fevers of unknown origin in the south of the country [27]. However, the current prevalence is still not well known, since reports show that most cases so far are sporadic. One of the few clinical studies conducted in northern Vietnam indicated that 40.9% and 33.3% of AUF patients in whom malaria, dengue fever and typhoid fever were excluded were infected with *O*. *tsutsugamushi* and *R*. *typhi*, respectively, based on serological tests [28]. Recently, a molecular epidemiological study described the presence of *O*. *tsutsugamushi*, including Karp (77%), TA763 (15.5%) and JG-v (7.5%) groups, in 13 isolates which had been collected from patients in 3 provinces in central Vietnam [9]. In addition, laboratory diagnostic tests are unavailable in almost all hospitals in the country and there are no epidemiological and clinical studies of other rickettsial diseases in Vietnam as of yet. Therefore, diagnosis and treatment can be considerably delayed when typical clinical signs such as rash and eschars are lacking or are not observed.

Quang Nam province is a coastal province in central Vietnam that may be a highly endemic area for *O*. *tsutsugamushi*, as well as other rickettsial infections. Despite this, cases are rarely documented. This study used standard diagnostic tools including immunofluorescence serology as well as emerging methods, such as molecular diagnostic testing on eschar swabs to diagnose cases of rickettsial infections in this area and provide information about the genotypes circulating there.

## Materials and methods

### Study design, site, entry criteria, and data collection

This descriptive study was conducted from March to September 2015 at the infectious diseases departments of three major general hospitals in Quang Nam province in Vietnam. These included Quang Nam Central General Hospital, Quang Nam Provincial Hospital and Northern Quang Nam Mountainous Hospital, which are all situated in the center of the country and have an approximate total of 1800 beds between them. These hospitals admit most of their patients from local health facilities around the province on a referral basis. Patients were included in this study if they fulfilled three primary criteria: 1) age ≥15 years; 2) axillary temperature ≥37.5°C; and 3) having had at least one of the following four secondary findings: eschar, skin rash, lymphadenopathy, hepatomegaly and/or splenomegaly. All patients were recorded the basic information of medical history and clinical manifestations but also the history of exposure to the forest within 30 days. One whole blood and one serum specimen were collected immediately from every patient. For the patients who presented with one or several eschars, swab samples were directly collected, by rotating vigorously, at the base of the eschar after removing the crust, as previously described [15]. The eschar lesions that were very dry were wetted with sodium chloride 0.9% before swabbing to obtain as much swabbed material as possible. All specimens were stored at -20°C until transportation and maintained at this temperature while under transport to Marseille.

### Ethics agreement

This study was approved by the ethical review committee of the University of Medicine and Pharmacy of Ho Chi Minh city and the Ministry of Health in Vietnam. All patients included in this study provided written informed consent.

### Laboratory tests

#### Serological testing

Specific microimmunofluorescence (IFA) assays were performed at the Research Unit on Infectious and Emerging Tropical Diseases (URMITE, Marseille, France) using whole-cell antigens from *O*. *tsutsugamushi* serotypes Karp, Kato, and Gilliam [29]. In addition, we also performed IFA for *Rickettsia typhi*, *Rickettsia felis*, *Rickettsia conorii*, and *Coxiella burnetii*. An IFA result in the acute phase was considered positive if positive antibody titers were >1:128 for immunoglobulin G (IgG) and 1:64 for immunoglobulin M (IgM) [30].

#### DNA extraction

For each patient, 200 μl of whole blood was used for DNA extraction and the remaining blood was stored at -20°C. Each eschar swab was immersed in 400 μL of G2 Buffer—a lysis buffer for use with EZ1 genomic DNA procedures—for 1 hour to release eschar materials and 200 μL of the supernatant was collected after a quick spin for DNA extraction. Genomic DNA was individually extracted from both the whole blood and eschar swab suspension using the DNA EZ1 extraction kit (Qiagen, Hilden, Germany) according to the manufacturer’s instructions. The DNA was then eluted in 100 μL of Tris EDTA (TE) buffer using the DNA-extracting EZ1 Advanced XL Robot (Qiagen). DNA was either immediately used or stored at -20°C until molecular analysis. A disinfection of the DNA-extracting EZI Advanced XL Robot was performed after each batch of extraction as per manufacturer’s recommendations to avoid cross-contamination.

#### Quantitative PCR

DNA samples were tested using PCR with genus-specific primers and probes targeting specific sequences of *Orientia tsutsugamushi*, *Rickettsia* spp., *Bartonella* spp., all *Anaplasmataceae*, *Borrelia* spp., and *Coxiella burnetii*. *Rickettsia felis* and *Rickettsia typhi* were also targeted in this study by specific qPCR systems. Real-time quantitative PCR was carried out according to the manufacturer’s protocol with a CFX Connect Real-Time PCR Detection System (Bio-Rad, Hercules, CA, USA) and the Eurogentec Takyon qPCR kit (Eurogentec, Seraing, Belgium). The periplasmic serine protease coding gene was used to detect *O*. *tsutsugamushi* [13], and *Rpr331* was used to detect *R*. *typhi* [31]. The guanosine coding gene was used to detect *R*. *felis* [32]. *gltA* was used for the spotted-fever group *Rickettsia* spp. [13]. ITS2 was used for *Bartonella* spp. [33] and *Borrelia rrs* was used for *Borrelia* spp. [34]. IS30a was used for *Coxiella burnetii* [35] and *rrl* was used for *Anaplasma* spp. [36]. *O*. *tsutsugamushi*, *R*. *montanensis*, *B*. *elizabethae*, *A*. *phagocytophilum*, *C*. *burnetii*, *B*. *crocidurae*, *R*. *felis* and *R*. *typhi* DNA was used as positive qPCR controls for the primers and probe, targeting respectively all *O*. *tsutsugamushi*, *Rickettsia*, *Bartonella*, *Anaplasma*, *Coxiella* and *Borrelia* species, *R*. *felis* and *R*. *typhi*. For each run, a DNA-free PCR mix was used as a PCR negative control.

#### Standard PCR and sequencing

DNA samples that were positive for *O*. *tsutsugamushi*-specific qPCR were submitted to conventional PCR amplification using a Bio-Rad Thermocycler (Bio-Rad Laboratories, Hercules, CA) prior to sequencing. Primers targeting an *O*. *tsutsugamushi 56kDa-TSA* gene fragment were used as previously described [37]. The DNA from *O*. *tsutsugamushi* was used as a PCR positive control, and a PCR mix without DNA as a negative control. Amplification products were controlled by electrophoresis with a 1.5% agarose-tris-borate-EDTA gel containing ethidium bromide. PCR products were sequenced using a Big Dye Terminator kit and an ABI PRISM 3130 Genetic Analyzer (Applied BioSystems, Courtaboeuf, France). The sequences were analyzed using the ABI PRISM DNA Sequencing Analysis software version 3.0 (Applied BioSystems) and compared to sequences available in the GenBank database using the BLAST algorithm (http://blast.ncbi.nlm.nih.gov/Blast.cgi). Sequences are available in GenBank at accession numbers from KU871377 to KU871390.

A phylogenetic analysis of *O*. *tsutsugamushi* genotypes on the basis of the sequences of 56-kDa TSA genes was conducted to determine the genetic relatedness of strains from previous isolates in Vietnam and strains from other countries. A total of 93 complete and partial 56-kDa gene nucleotide sequences were retrieved from GenBank and used in the analysis. Sequences were aligned using CLUSTALW, and phylogenetic inferences obtained using the Bayesian phylogenetic analysis with TOPALi 2.5 software (Biomathematics and Statistics Scotland, Edinburgh, UK) within the integrated MrBayes application, using the Hasegawa-Kishino-Yano substitution model. Numbers at the nodes are percentages of bootstrap values obtained by repeating the analysis from 100-time replicates to generate a majority consensus tree (only those that are ≥80 were retained). The final set includes 372 base pairs.

### Statistical analysis

Categorical variables were summarized as frequencies and percentages. Different groups were compared using the Fisher exact test. The Student t-test and Mann-Whitney U-test were used to compare different continuous variable groups. Analysis was performed using SPSS 16.0 (2007, Chicago, SPSS Inc).

## Results

### Patient demographic and clinical information

**Fig 1:**
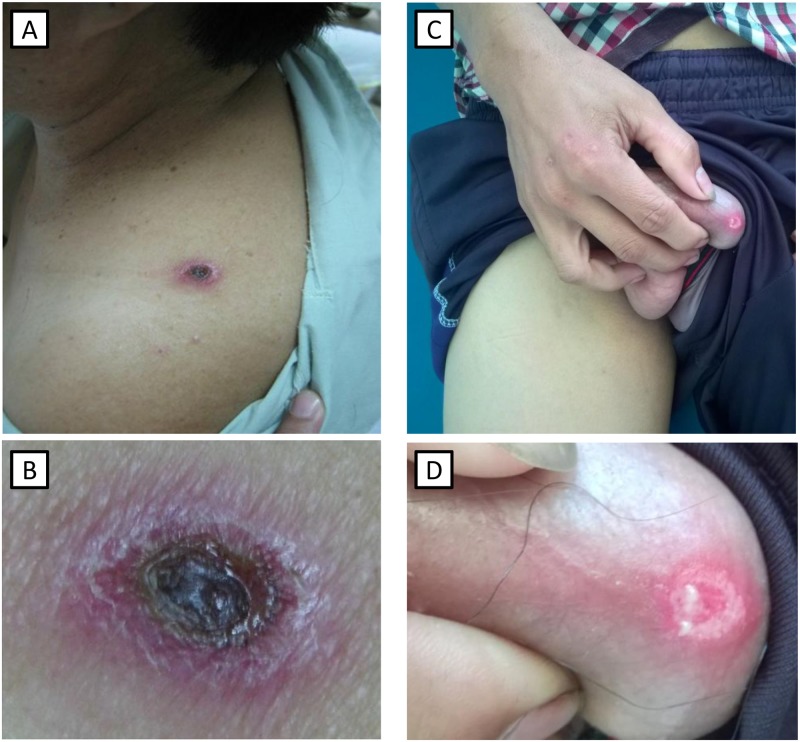
Eschars in scrub typhus patients. Eschar on the shoulder (a, b) of a female and on the base of the penis (c, d) of a male scrub typhus patient.

A total of 67 patients were enrolled and their clinical information is described in Table 1 and Fig 1. The median (range) age of these patients was 41 (16–74), and 39 of them (58.2%) were male. More than three-quarters (78.5%) of the patients were farmers. There were no significant differences in median age and job between scrub typhus group and the group of patients who tested negative for scrub typhus and murine typhus (non-scrub typhus/non-murine typhus group). However, 37.5% of patients in the scrub typhus group were male, whereas in the remaining group males comprised 75.8%. The median illness duration was one week, but some patients had been admitted more than 10 days after the fever. The proportion of patients who had worked or traveled in the forest was 48.5% overall, but only 38.7% in the scrub typhus group. Eschar lesions occurred in 18 patients (56.2%) of the scrub typhus group and 4 patients (12.1%) in the non-scrub typhus/non-murine typhus group. Headache was a common symptom in the patients, especially in the scrub typhus group (up to 90%). Patients in the scrub typhus group were significantly more likely to have lymphadenopathy (48.4% vs. 21.2%). Nausea and hepatomegaly were less frequent in both of these groups. No patient was observed to have a rash. There was a slight decrease in platelets with a mean platelet count of 146 ± 66 x10^9^/L in the scrub typhus group and 158 ± 72 x10^9^/L in the non-scrub typhus/non-murine typhus. However, the aminotransferases (AST, ALT) increased from 3 to 5 fold in comparison with normal range. The median of serum AST level was 104 (26–435) IU/L and 91 (20–438) IU/L and serum ALT level was 76.5 (16–420) IU/L and 80 (26–323) IU/L in scrub typhus and non-scrub typhus/non-murine typhus, respectively.

**Table 1:** Clinical features of scrub typhus patients and non-scrub typhus non-murine typhus patients.

Features	Scrub typhus cases (n = 32)	Non scrub typhus/non-murine typhus cases (n = 33)	All of patients (n = 67)*
Age (y), ¥	42.5 (17–74)	38 (24–69)	41 (16–74)
No. (%) male	12 (37.5)	25 (75.8)	39 (58.2)
No. (%) farmer	26 (83.9)†	25 (78.1)†	51 (78.5)‡
Duration of fever (d)¥	7 (3–10)†	5 (2–11)	7 (2–11)†
Exposure to forest (%)	12 (38.7)†	20 (60.6)	32 (48.5)†
Chills (%)	11 (61.1)€	13 (65)₹	25 (64.1)£
Eschar (%)	18 (56.2)	4 (12.1)	22 (32.8)
Rash (%)	0	0	0
Lymphadenopathy (%)	15 (48.4)†	7 (21.2)	22 (33.3)†
Headache (%)	27 (90)‡	22 (66.7)	51 (78.5)‡
Nausea (%)	2 (6.5)†	2 (6.1)	4 (6.1)†
Hepatomegaly (%)	2 (6.5)†	2 (6.1)	4 (6.1)†
Splenomegaly (%)	3 (9.7)†	0	3 (4.5)†
Leukocyte count (x10^9^/L)¥	7.5 (4–21)‡	7.6 (2–11)	7.5 (2–21)‡
Platelets (x10^9^/L)ᶲ	146 (66)‡	158 (72)	154 (70)‡
Serum AST (IU/L) ¥	104 (26–435)‡	91 (20–438)	96 (20–438)‡
Serum ALT (IU/L) ¥	76.5 (16–420)‡	80 (26–323)	77 (16–420)‡
Duration of afebrile (d)¥⁑	2 (1–5)†	2 (1–5)	2 (1–5)†
No. day of Doxycycline (d)¥	5.5 (3–7)†	5 (4–7)	5 (3–7)†

*including 2 murine typhus cases;

^†^ 1 missing case;

^‡^ 2 missing cases;

^₹^ 13 missing cases;

14 missing cases;

^£^ 28 missing cases;

^¥^ Median (range);

^ᶲ^ Mean (SD)

^⁑^ Afebrile was defined as normal body temperature (under 37.5°C) without using any antipyretics. *Duration of afebrile* was counted from first specific antibiotic time-point to afebrile time-point.

### Laboratory test results

A total of 32 patients (47.8%) were confirmed as having *O*. *tsutsugamushi* infection by either qPCR or IFA. Six cases (28.1%) tested positive by both qPCR and IFA. Fourteen cases (43.8%) were positive for qPCR only, and 12 (37.5%) cases were positive for only IFA.

Among the 20 cases who tested positive by *O*. *tsutsugamushi* qPCR, 12 (60%) tested positive using their eschar only; 5 (25%) had both their blood and eschars positive. Also, the blood of 3 additional patients (15%) tested positive. No eschar was found on these patients.

For the 18 cases that were positive for *O*. *tsutsugamushi* by IFA, serum specimens were tested by separate IFA for IgG and IgM antibodies to each bacterial antigen. The antibody titers of Gilliam antigen ranged from 128 to 8192, with a median of 128 and a geometric mean titer (GMT) of 370.5. Antibody titers ranged from 128 to 4096 (median: 128) for both Kato and Karp genotypes. However, GMT values were 282.6 and 308 for Karp and Kato genotypes respectively.

A total of 14 quality sequences of a 411 base pair portion of the TSA 56 kDa type-specific gene were obtained including 12 sequences from eschar swab specimens and 2 sequences from the whole blood of two patients on who no eschar was noticed.

**Fig 2:**
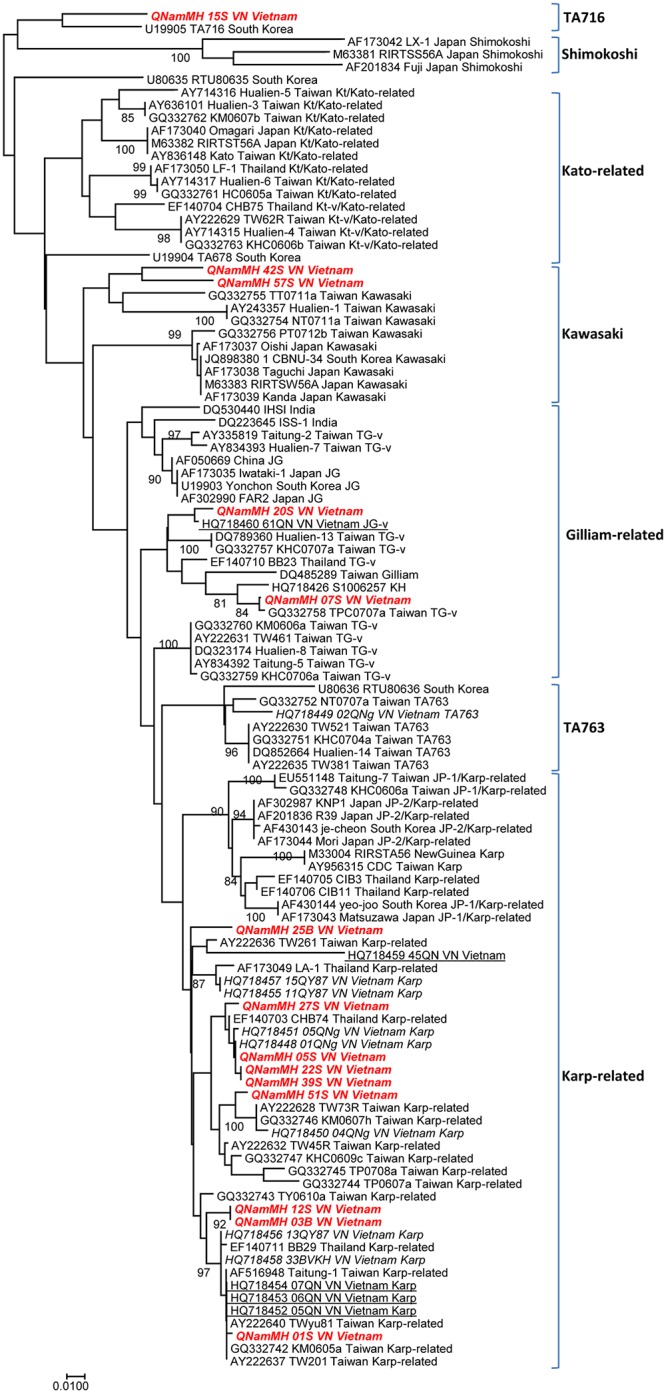
Phylogenetic tree of *O*. *tsutsugamushi*, based on 56-kDa TSA gene sequences. A consensus phylogenetic tree showing the relationships of studied strains of *O*. *tsutsugamushi*, based on 56 kDa TSA gene sequence comparison. GenBank accession numbers are indicated at the beginning. Sequences were aligned using CLUSTALW, and phylogenetic inferences obtained using the Bayesian phylogenetic analysis with the TOPALi 2.5 software (Biomathematics and Statistics Scotland, Edinburgh, UK) within the integrated MrBayes application, using the Hasegawa-Kishino-Yano substitution model. Numbers at the nodes are percentages of bootstrap values obtained by repeating the analysis from 100-time replicates to generate a majority consensus tree (only those that are ≥80 were retained). The final set includes 372 base pairs. The scale bar represents a 1% nucleotide sequence divergence. Reference strains from GenBank are identified by their accession number. Sequences from Quang Nam province are presented in bold and italic (this study) and underlined (Duong V. study). Sequences from other provinces in Vietnam are presented in italics (Duong V. study).

Genetic analysis of the 56-kDa TSA gene sequences demonstrated that the 14 sequences obtained in this study were related to 4 strains, including Karp, Kawasaki, TA716 and Gilliam (JG-v and TG-v) (Fig 2 and Table 2). The majority (9/14; 64.4%) of contemporary *O*. *tsutsugamushi* genotypes were related to the Karp strain, with a percentage nucleotide identity (PNI) ranging from 97% to 100% in comparison to Karp strain references (Tables 2 and 3). Two (2/14; 14.2%) sequences demonstrated the highest similarity to the Kawasaki strain, with a PNI ranging from 90% to 93% when compared to the Kawasaki reference strain GQ332755. The QNamMH_15S_VN sequence (1/14; 7.1%) was most closely related to the TA716 strain U19905 (PNI: 91%). Two remaining sequences (2/14; 14.2%) were related to Gilliam, in which one sequence was similar to the JG-v strain HQ718460 (PNI: 99%), and another one was similar to the TG-v strain GQ332758 (PNI: 100%).

**Table 2:** Genotype classification of *O*. *tsutsugamushi* from Quang Nam province, based on sequencing using the 56-kDa gene.

Patient	District	Type of specimen analyzed	Serological diagnosis				Gen Bank accession No. of 56-kDa TSA	Group	Isolation/56-kDa gene references
			Day of serum collection (after onset)	IgG/IgM titers					
				Gilliam	Kato	Karp			
QNamMH_01S_VN	Dai Loc	Eschar	5	256/0	256/0	256/0	KU871377	Karp 99%	AY222640
QNamMH_05S_VN	Phu Ninh	Eschar	6	64/0	64/0	64/0	KU871378	Karp 100%	HQ718448
QNamMH_07S_VN	Nui Thanh	Eschar	7	Neg	Neg	Neg	KU871379	TG-v 100% Gilliam-related	GQ332758
QNamMH_12S_VN	Dai Loc	Eschar	5	Neg	Neg	Neg	KU871380	Karp 98%	HQ718456
QNamMH_15S_VN	Nui Thanh	Eschar	4	Neg	Neg	Neg	KU871381	TA716 91%	U19905
QNamMH_20S_VN	Nui Thanh	Eschar	8	128/0	128/0	128/0	KU871382	JG-v 99% Gilliam-related	HQ718460
QNamMH_22S_VN	Nui Thanh	Eschar	7	Neg	Neg	Neg	KU871383	Karp 99%	HQ718448
QNamMH_27S_VN	Phu Ninh	Eschar	7	Neg	Neg	Neg	KU871384	Karp 99%	HQ718451
QNamMH_39S_VN	Nui Thanh	Eschar	10	Neg	Neg	Neg	KU871385	Karp 99%	HQ718448
QNamMH_42S_VN	Tra My	Eschar	5	Neg	Neg	Neg	KU871386	Kawasaki 93%	GQ332755
QNamMH_51S_VN	Nui Thanh	Eschar	5	Neg	Neg	Neg	KU871387	Karp 98%	AY222628
QNamMH_57S_VN	Nui Thanh	Eschar	10	128/0	128/0	128/0	KU871388	Kawasaki 90%	GQ332755
QNamMH_03B_VN	Nui Thanh	Whole blood	7	128/0	128/0	128/0	KU871389	Karp 98%	HQ718456
QNamMH_25B_VN	Phu Ninh	Whole blood	8	Neg	Neg	Neg	KU871390	Karp 97%	HQ718455

**Table 3:** Prevalence of current circulating genotypes of *O*. *tsutsugamushi* in humans in Vietnam.

Genotypes	Places	This study, 2016 n (%)	Duong, V., et al., 2013 [9] n (%)	Combination of two studies n (%)
Karp	Quang Nam	9 (64.4)	3 (60)	12 (63.2)
	Central Vietnam*	-	10 (77)	19 (70.4)
Kawasaki	Quang Nam	2 (14.3)	-	2 (10.5)
	Central Vietnam	-	-	2 (7.4)
TA716	Quang Nam	1 (7.1)	-	1 (5.3)
	Central Vietnam	-	-	1 (3.7)
TA763	Quang Nam	-	1 (20.0)	1 (5.3)
	Central Vietnam	-	2 (15.5)	2 (7.4)
JG-v Gilliam related	Quang Nam	1 (7.1)	1 (20.0)	2 (10.5)
	Central Vietnam	-	1 (7.5)	2 (7.4)
TG-v Gilliam related	Quang Nam	1 (7.1)	-	1 (5.3)
	Central Vietnam	-	-	1 (3.7)

*Three provinces, including Quang Nam, Quang Ngai and Khanh Hoa in the study of Duong V.

Two patients (3.0%) tested positive for murine typhus with *Rickettsia typhi* IFA positive results. qPCR tests were negative. There were no specimens from any of the patients positive for *R*. *felis*, SFG *Rickettsia*, *C*. *burnetii*, *Bartonella* spp., *Borrelia* spp. and *Anaplasma* spp. with qPCR.

## Discussion

Our study is one of the few using eschar swabs to diagnose rickettsial diseases, and it is also the first study to utilize this type of specimen for scrub typhus diagnosis in Vietnam.

The prevalence of eschars in patients diagnosed with scrub typhus has been reported as between 7%-80% [38]. The proportion of eschars in scrub typhus in our study was similar to the report of Hamaguchi et al. in northern Vietnam (56.2% and 62.9%, respectively [28]). We found an eschar in all cases of Gilliam, Kawasaki and TA716 groups, but in only 7 (77.8%) cases of Karp. These results are consistent with previous reports highlighting the variability in eschar presence depending on the *O*. *tsutsugamushi* genotype [39]. In South Korea, Kim et al. (2011) found that 97% patients infected with the Boryoung cluster presented eschars whereas 73.7% presented eschars with the Karp cluster [39]. Similarly, lymphadenopathy was only present in 5 (55.5%) cases of Karp in our study, whereas it was present in all cases caused by other genotypes. Also, no rash was found in any of our confirmed cases of scrub typhus. Skin rash is reported to be present in approximately 45–50% of patients with scrub typhus [4, 40]. However, its frequency seems to vary depending on the area. Its frequency is high in Japanese patients (93%) [41], but low in Thai patients (7%) [42] even rarer in Indian patients (1.7%) [43]. Recently, a few studies paid attention to the differences of clinical features according to genotypes. Rash was found less frequently associated with Karp genotype (68.4%) in comparison to Boryoung genotype (94%) in South Korea [39]. Karp genotype (38.1%) was also found less frequently associated with rash than TA763 genotype (100%) in China, but more frequently associated with rash than Kato (11.1%) and Gilliam (20%) [44]. Thus, the predominance of Karp genotype might explain the absence of rash in our study [39]. However, a rash could have also been missed since the relatively dark-skin of Vietnamese patients or rash might be absent the day of the clinical examination at hospital. Due to our limited sample size, further investigation should be conducted on this issue.

PCR using eschar swabs has been used successfully for the diagnosis of infections caused by tick-borne spotted-fever group rickettsiae including *R*. *africae* [12], *R*. *conorii conorii*, *R*. *sibirica mongolitimonae*, *R*. *slovaca* and *R*. *australis* [13]. In scrub typhus studies, Kim et al. (2006) reported that the sensitivity of PCR on eschar swabs was 86% and the specificity was 100% [14], whereas the sensitivity of PCR on blood has been known to be lower (29%) [45]. In addition, swab samples are preferred to biopsy samples and blood samples, since the swabbing of an eschar is noninvasive, easy and painless. The sensitivity of qPCR method on eschar swabs could not be evaluated in our study. However, we found a high rate of positive results of the qPCR using eschar specimens, with 17 positive samples for *O*. *tsutsugamushi* in 20 eschars specimens (85%) obtained from patients. These positive specimens were sequenced and resulted in 12 good quality sequences. On the other hand, the three patients with eschar who were tested negative for *O*. *tsutsugamushi* by qPCR using eschar swab might not be scrub typhus since qPCR on blood and IFA were also negative. These cases might however be scrub typhus but our clinical doctors might have collected insufficient eschar material during swabbing. In case dry eschar lesions are observed, we wet the lesions to collect enough material as previous described [46]. Additionally, the day of serum sample collection could lead to negative IFA results if collection occurs during the early stages of infection. We also lacked convalescent serum and therefore we could not confirm *O*. *tustsugamushi* infection in these 3 cases.

**Fig 3:**
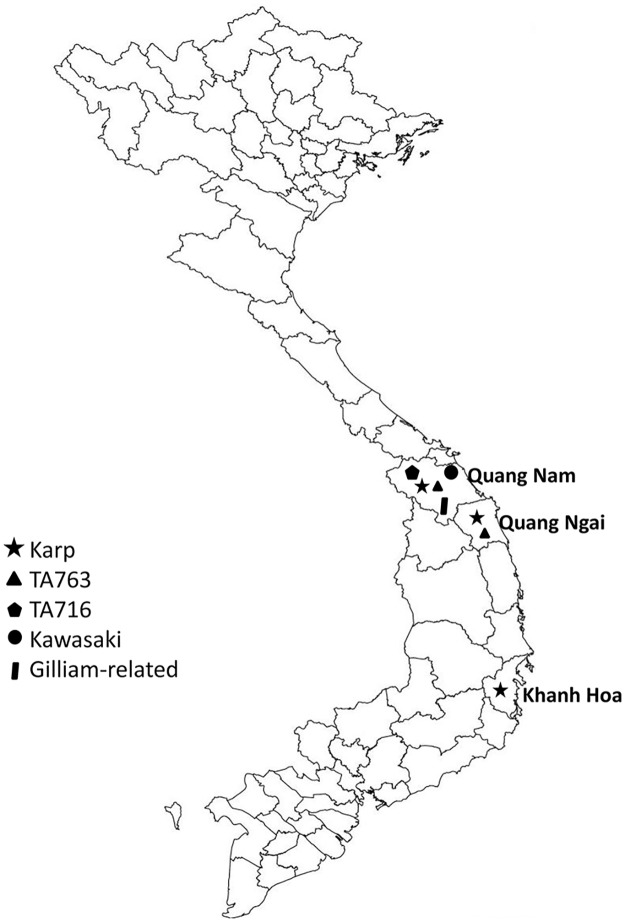
Geographic distribution of the genotypes of *Orientia tsutsugamushi* in humans in Vietnam.

Our work provides more evidence of the genetic diversity of *O*. *tsutsugamushi* in Vietnam, since 4 genotypes were described in our study conducted in Quang Nam. In 2013, Duong et al. reported 3 genotypes of *O*. *tsutsugamushi*, including Karp (77%), TA763 (15.5%) and JG-v (17.5%), in 3 provinces of central Vietnam, including Quang Nam (Fig 3) [9]. Among the 4 genotypes that were found in our study, Karp also predominated (9/14; 64.4%). Our results indicate greater genetic diversity in this province, with 3 new genotypes (Kawasaki, TA716 and Gilliam-related) (Table 3). We cannot determine how the genetic diversity of *O*. *tsutsugamushi* in our research area compares with that in other regions of Vietnam. However, the current data for the bacterial genotypes in Vietnam are from central Vietnam and now include 5 different genotypes among 27 sequences (14 sequences of this study and 13 previous sequences [9]) (Fig 3 and Table 3). The most common group is Karp (19/27; 70.4%). The second most prevalent one is Gilliam (3/27; 11.1%), and less common groups are TA763 (2/27; 7.4%), Kawasaki (2/27; 7.4%) and TA716 (1/27; 3.7%). Geographically, the Karp-related group represents the most frequent genotype of *O*. *tsutsugamushi* in Japan, Korea, China, and Southeast Asia [47].

Our findings may be useful in accuracy of diagnostic tests in the endemic area. The performance of diagnostic tools can be dependent upon the genotype of the bacteria [48]. In our study, patients infected with genotypes like TA716, TG-v and Kawasaki tested negative by IFA using Gilliam, Kato and Karp antigen. Therefore, the combination of the results from this study and the previous study in Vietnam [9], we suggest that polyclonal antigen pools used for serological testing in the future should contain at least Karp, Kawasaki, TA716, TA763 and Gilliam (JG-v and TG-v) antigens for Vietnamese patients, as well as patients who have traveled to Vietnam. This may also provide more information of *O*. *tsutsugamushi* genotypes in this area to enable the development of a vaccine against scrub typhus in the future. Indeed, vaccine development is necessary for scrub typhus and was initiated last century, but attempts have not been successful yet [49]. One of the barriers is the diversity of *O*. *tsutsugamushi* strains and those strains do not stimulate an effective cross-protective immune response [49]. Therefore, our finding could be included in the multinational dataset of *O*. *tsutsugamushi* antigens [50] that will be used to do further research on vaccination development.

Two cases of murine typhus have also been diagnosed in our study. Murine typhus, caused by *Rickettsia typhi* and transmitted by fleas, has been reported throughout the world, notably in the tropics [51]. A pilot meta-analysis which selected acute febrile illness studies from every continent except Antarctica, indicated that the median proportion of murine typhus was 7.9% (IQR 4.2–14.5%) [51]. In Southeast Asia, murine typhus is an important cause of febrile illness, reported in 9.6% [52], 6.1% [53] and 7.1% [54] of undifferentiated fever patients in Laos, Thailand and Indonesia, respectively. Most recently, Hamaguchi et al. (2015) used IFA testing to detect *R*. *typhi*, and found that among patients with acute undifferentiated fever in hospitalized patients in Northern Vietnam, 33.3% were confirmed to have murine typhus infection [28]. Our two cases presented with non-specific symptoms, such as headache and fever, but no rash or eschar; thus, the clinicians did not suspect murine typhus, as is frequently the case. Patients with acute undifferentiated fever patients should definitely be screened for murine typhus and further studies are needed in this region.

Spotted-fever group rickettsiae (SFGR) have been reported throughout the world in recent decades [55]. *Rickettsia felis*, a member of SFGR, is now considered an emerging infection globally [55, 56] and has been reported in some neighboring countries of Vietnam, such as Laos [52] and Thailand [57]. It is transmitted by fleas and may also be transmitted by mosquitoes [58]. Some tick-borne SFGR have been found in China, such as *Rickettsia heilongjiangensis* and *Rickettsia sibirica* [56]. In our patients, SFGR was not detected. SFGR infections may be present sporadically and need to be surveyed by further studies, which should contain larger numbers of acute febrile samples. Similarly, *Coxiella burnetii*, the etiological agent of Q fever, was not identified in our patients although it has a worldwide distribution and has been reported in some countries near Vietnam such as China and Thailand [59].

One limitation of our study was a lack of convalescent serum specimens, which might have helped us detect more murine typhus or other rickettsial infections, especially when molecular and serological test results were negative in the acute febrile phase.

In conclusion, eschar should be routinely considered for molecular diagnosis of scrub typhus in endemic patients as well as in travelers, since it is very useful for rapid detection of *Orientia tsutsugamushi* and helps to accurately diagnose scrub typhus in its early phase. In addition, the serological test for *Rickettsia typhi* should be used to diagnose AUF patients, and convalescent serum specimens also need to be included for diagnosis and research on AUF in Quang Nam province, as well as throughout Vietnam in the future.

## Supporting information

S1 Table Sequences of the qPCR primers and probes used in this study. (DOCX)

S1 Checklist STARD checklist. (PDF)
