# Expression of Concern: Use of eschar swabbing for the molecular diagnosis and genotyping of *Orientia tsutsugamushi* causing scrub typhus in Quang Nam province, Vietnam

**DOI:** 10.1371/journal.pntd.0010975

**Published:** 2022-12-13

**Authors:** The PLOS Neglected Tropical Diseases Editors

This article [1] has been identified as one of a series of submissions for which we have concerns about the reported research ethics approval information and the article’s adherence to PLOS research ethics policies.

PLOS will be investigating these concerns in accordance with COPE guidance and journal policies. Meanwhile, the *PLOS Neglected Tropical Diseases* Editors issue this Expression of Concern.
