# Correction: Use of eschar swabbing for the molecular diagnosis and genotyping of *Orientia tsutsugamushi* causing scrub typhus in Quang Nam province, Vietnam

**DOI:** 10.1371/journal.pntd.0012563

**Published:** 2024-10-07

**Authors:** The PLOS Neglected Tropical Diseases Editors

The *PLOS Neglected Tropical Diseases* Editors issue this notice to resolve the concerns underlying the previously published Expression of Concern on this article [1, 2].

The issues underlying the Expression of Concern [2] included that the article [1] did not report ethics approval from the corresponding author’s home country (France) or the Vietnamese ethics approval number(s) and date(s).

A representative from the Aix-Marseille Université stated that the institutional investigation into the ethics concerns concluded this article meets ethical standards. They stated that sample collection took place in Vietnam and French researchers were not involved in the sample collection process, so French ethics approval was not required. They also noted that the French contribution consisted exclusively of analyzing samples received from Vietnam.

The authors provided documents #1645/QD-BV and #48/CN-BDGDD for editorial review, and explained that the approval from the Quang Nam Central General Hospital covers collection of samples from all three hospitals involved in the study. The approvals, for which details are provided in the updated Ethics statement below, are for research into epidemiology, pathology, and clinical characteristics of Rickettsia* infection in Quang Nam.

*Orientia tsutsugamushi, investigated in [1], belongs to the Rickettsia infection group. It is in the Rickettsiales order and Rickettsiaceae family, and was previously classified in the *Rickettsia* genus [3].

In light of the documentation and responses provided, the Ethics statement in the Materials and methods section of [1] is updated to:

This study was approved by the Ministry of Health, Quang Nam Central General Hospital (#1645/QD-BV issued on 09 December 2014), and the Ministry of Health Evaluation Committee on Ethics in Biomedical Research in Vietnam (#48/CN-BDGDD issued on 14 July 2015). All patients included in this study provided written informed consent.

With this update, the *PLOS Neglected Tropical Diseases* Editors consider the ethics approval concerns resolved. This Correction supersedes the prior Expression of Concern [2].
