# Supplementary material for: Use of eschar swabbing for the molecular diagnosis and genotyping of Orientia tsutsugamushi causing scrub typhus in Quang Nam province, Vietnam
Source: PLoS Negl Trop Dis. 2017 Feb 27;11(2):e0005397. doi: 10.1371/journal.pntd.0005397 (PMC5344524; doi:10.1371/journal.pntd.0005397)
Supplement: S1 Table — (DOCX) [file pntd.0005397.s001.docx]

**Supplement 1. The primers and probes used for each of the targets in this study**

| **TARGETED ORGANISM** | **GENE NAME** | **PRIMER** | | **SEQUENCES** |
| --- | --- | --- | --- | --- |
| *Orientia tsutsugamushi* | Periplasmic serine protease | Forward | TSULC_F | **AACTGATTTTATTCAAACTAATGCT** |
|  |  | Reverse | TSULC_R | **TATGCCTGAGTAAGATACRTGAATRGAATT** |
|  |  | Probe | TSULC_P | **6FAM-TGGGTAGCTTTGGTGGACCGATGTTTAATCT** |
| *Rickettsia felis* | Guanosine | Forward | Rfelg_F | **GCATATACTTTATTGTGCGCAAGTT** |
|  |  | Reverse | Rfelg_R | **TTTATCGATTGACAGAAGAAGAAATCA** |
|  |  | Probe | Rfelg_P | **6-FAMTCGCTTTTTGGGATTGTTTGCCAGA** |
| *Rickettsia typhi* | Hypothetical protein | Forward | Rpr_274_F | **TGTCAGATTATAAAGACGATGCTCAGA** |
|  |  | Reverse | Rpr_274_R | **GCAGCTTGTACTCCTTTAATTTGTTC** |
|  |  | Probe | Rpr_274_P | **6FAM- CCGCTACCGCAAATCCATCAGA** |
| *Rickettsia* spp. | gltA | Forward | RKND03_F | **GTGAATGAAAGATTACACTATTTAT** |
|  |  | Reverse | RKND03_R | **GTATCTTAGCAATCATTCTAATAGC** |
|  |  | Probe | RKND03 P | **6FAM- CTATTATGCTTGCGGCTGTCGGTTC** |
| *Anaplasmataceae* | 23S | Forward | TtAna_F | **TGACAGCGTACCTTTTGCAT** |
|  |  | Reverse | TtAna_R | **GTAACAGGTTCGGTCCTCCA** |
|  |  | Probe | TtAna_P | **6FAM- GGATTAGACCCGAAACCAAG** |
| *Bartonella* spp. | *ITS* | Forward | Barto_ITS2_F | **GGGGCCGTAGCTCAGCTG** |
|  |  | Reverse | Barto_ITS2_R | **TGAATATATCTTCTCTTCACAATTTC** |
|  |  | Probe | Barto_ITS2_P | **6FAM- CGATCCCGTCCGGCTCCACCA** |
| *Borrelia* spp. | *16S* | Forward | Bor_16S_3F | **AGCCTTTAAAGCTTCGCTTGTAG** |
|  |  | Reverse | Bor_16S_3R | **GCCTCCCGTAGGAGTCTGG** |
|  |  | Probe | Bor_16S_3P | **6FAM- CCGGCCTGAGAGGGTGAACGG** |
| *Coxiella burnetii* | Hypothetical protein | Forward | CB_IS30A_3F | **CGCTGACCTACAGAAATATGTCC** |
|  |  | Reverse | CB_IS30A_3R | **GGGGTAAGTAAATAATACCTTCTGG** |
|  |  | Probe | CB_IS30A_3P | **6FAM- CATGAAGCGATTTATCAATACGTGTATGC** |
